# Widefield frequency-domain autofluorescence lifetime imaging for detecting breast cancer in murine xenograft tumor tissues

**DOI:** 10.1117/1.JBO.30.S2.S23911

**Published:** 2025-09-12

**Authors:** Oscar R. Benavides, Lakhvir Singh, Samantha Morganti, Melody Yeh, Tabassum A. Tasmi, Anna Jovanovic, Rachel Wellner, Alex J. Walsh

**Affiliations:** a Texas A&M University, Department of Biomedical Engineering, College Station, Texas, United States; b Caelum Diagnostic Solutions, Venice, California, United States

**Keywords:** autofluorescence, fluorescence lifetime imaging, breast cancer detection, cellular metabolism, optical diagnostics

## Abstract

**Significance:**

Early and accurate detection of breast cancer is important for effective treatment and improved patient outcomes.

**Aim:**

We demonstrate a custom widefield frequency-domain fluorescence lifetime imaging (WF-FLIM) system for label-free imaging of cancerous and noncancerous mammary tissue.

**Approach:**

A custom multispectral WF-FLIM microscope was designed and built to image the endogenous fluorophores nicotinamide adenine dinucleotide (NADH) and flavin adenine dinucleotide (FAD) for identifying the metabolic signatures of cancerous and noncancerous tissue. The lifetime measurements of the system were validated with images of NADH and FAD solutions. WF-FLIM images were acquired for MDA-MB-231 and BT474 cells and freshly excised tissues of breast cancer xenograft tumors.

**Results:**

The WF-FLIM images of different concentrations of free and bound NADH and FAD solutions confirmed the isolation of NADH and FAD signals and different lifetime values for free and protein-bound NADH. Analysis of the WF-FLIM images of breast cancer samples revealed that the mean phase and modulation lifetimes were shorter for the metastatic MDA-MB231 tumors as compared with the nonmetastatic BT-474 tumors and noninjected control mammary tissues.

**Conclusions:**

We demonstrate the utility of widefield frequency domain FLIM to rapidly measure autofluorescence lifetime differences across cells and tissues.

## 1 Introduction

Breast cancer accounts for nearly a third of all new cancer diagnoses for women in the United States.^1^ The American Cancer Society estimates the rate of breast cancer diagnosis alone will grow by nearly 30% by 2040.^2^ Timely diagnosis is a fundamental tenet of the National Academy of Medicine and the National Cancer Policy Board, as this can reduce time-to-treatment, patient anxiety, and ultimately improve patient outcomes.^3^ The standard of care for cancer diagnosis involves acquiring a tissue biopsy of the suspicious lesion for histopathological assessment. This process requires a core needle biopsy of the lesion to harvest the tissue, which is then transported to a histopathology lab where it is fixed, processed, embedded, sliced, stained, visualized, and analyzed by a pathologist. A 2021 study by the College of American Pathologists found that less than 60% of routine biopsy pathology diagnoses were performed in an adequate turnaround time (TAT) of less than two business days.^4^ Unfortunately, biopsy tissue histopathology analysis is a time-consuming process that can take up to 2 weeks for results.^5^ This delay can be longer, up to 4 months, for medically underserved populations (MUPs) with limited access to traditional histopathology labs.^6^^–^^8^

Pathology lab TAT can vary due to sample burden, staff availability and expertise, distance between hospital and lab, and sample processing steps such as overnight fixation, re-embedding, re-cutting, or special immunostains.^9^^–^^11^ Further, to reduce the likelihood of additional diagnostic delay due to discordant pathology, clinicians will often deliberately oversample the suspicious tissue and harvest five or more biopsies.^12^^–^^16^ Oversampling of biopsies subjects patients to additional painful procedures and potential adverse events such as bleeding and infection.^17^^–^^19^ Pathology lab efficiency, challenged by the growing volume of biopsy tissues requiring analysis, could benefit from a diagnostic device that provides faster results than traditional H&E staining-based analysis. Specifically, a diagnostic tool that does not require special tissue handling or processing, while accurately distinguishing between cancerous and benign tissue, would minimize pathology TAT and improve patient outcomes. Such a device could be employed at the point-of-care (POC) to verify the diagnostic yield of biopsied tissue, minimizing the need for tissue oversampling and reducing the risk of potential adverse events.

Fluorescence lifetime imaging microscopy (FLIM) is an optical imaging technique that measures the fluorescence lifetime of a sample.^20^ Fluorescence lifetime is the average time a fluorophore spends in the excited state before relaxing to the ground state and emitting a photon. The lifetime of a molecule is sensitive to local microenvironment factors such as quenching, pH, ion concentration, and molecular interactions.^21^ FLIM of the endogenous and autofluorescent molecules, nicotinamide adenine dinucleotide (NADH) and flavin adenine dinucleotide (FAD), can quantify cell metabolism, disease progression, and tumor-drug response.^22^^–^^25^ Autofluorescence lifetime imaging microscopy (aFLIM) traditionally has required the use of expensive multiphoton (MP) light sources and time-correlated single-photon counting (TCSPC) detectors. aFLIM has faced challenges in translating to the clinic due to expensive costs, bulky equipment, slow analysis speed, and the expertise required to operate and maintain MP-TCSPC systems. Frequency domain FLIM (FD-FLIM) offers an alternative to time-domain FLIM (TD-FLIM) methods.^20^ In FD-FLIM systems, the sample is excited using intensity-modulated light and the fluorescence emission, which is shifted in both phase and modulation depth, is then demodulated to extract the phase and modulation lifetimes, respectively. The phase and modulation lifetimes are independent measures and are useful in understanding the multiple lifetime components for the same spatial location. FD-FLIM provides several advantages over traditional MP-TCSPC TD-FLIM for POC metabolic imaging-based diagnosis because it (1) only requires an intensity-modulated light source and gain-modulated detector allowing for a small and portable footprint and (2) can utilize a camera for fast widefield (WF) imaging. In addition, novel and large single-photon avalanche diode (SPAD) array detectors enable widefield FD-FLIM (WF-FLIM) as they offer enhanced low-light (i.e., autofluorescence) and high-speed gating performance compared with standard sCMOS cameras.^26^^–^^29^ To date, WF-FLIM of NADH and FAD for detecting cancer in freshly harvested biopsy tissues has not been explored or realized.

Here, a custom WF-FLIM microscope was designed and evaluated for rapid autofluorescence lifetime imaging of tissue samples. WF-FLIM was performed on excised murine xenograft tumor tissues, generated using athymic mice injected with either metastatic (MDA-MD-231) or nonmetastatic (BT474) human breast cancer cells. The results demonstrate that autofluorescence WF-FLIM can distinguish between nontumor and tumor tissues, and between metastatic and nonmetastatic cells and tumors in freshly harvested tissues. The WF-FLIM examination of the tissues was compatible with subsequent histopathological analysis. This proof-of-concept study exemplifies the use and utility of a desktop-sized, widefield FD-FLIM microscope for detecting cancer in biopsy tissues for aiding and improving breast cancer detection.

## 2 Materials and Methods

### 2.1 WF-FLIM Microscope Configuration

**Fig. 1:**
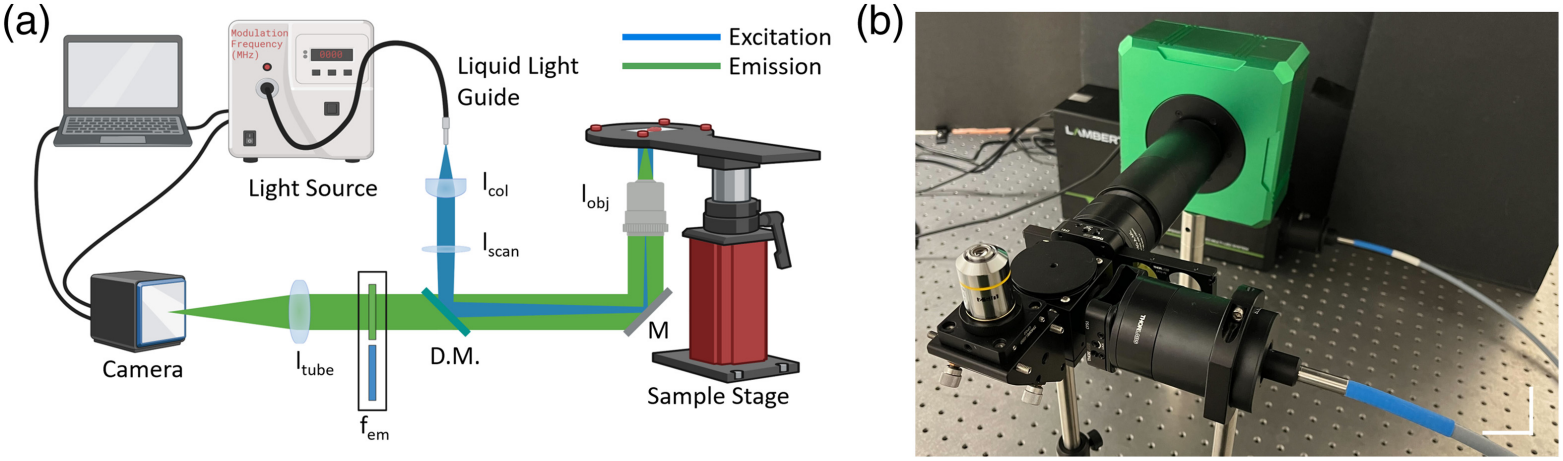
(a) Schematic and (b) photograph of the WF-FLIM microscope system. $l_{\mathrm{col}}=\text{collimating lens}$; $l_{\text{scan}}=\text{scan lens}$; D.M. = dichroic mirror; M = mirror; $l_{\mathrm{obj}}=\text{objective lens}$; $f_{\mathrm{em}}=\text{emission filter}$; $l_{\text{tube}}=\text{tube lens}$. Scale bar = 100 mm.

The WF-FLIM microscope was built using off-the-shelf components (Fig. 1). A 368-nm LED (multi-LED, Lambert Instruments) was used for fluorescence excitation and was focused with an aspheric lens (LLG3A4-A, Thorlabs) into a liquid light guide (LLG3-4T) and then collimated with an aspheric lens (LLG3A1-A, Thorlabs). The illumination light was focused by a scan lens (ACT508-200-A, Thorlabs) to the back focal plane of the objective lens (RMS10x, Thorlabs; 20X XLUMPLFLN, Olympus). The objective lens was positioned in an inverted epi-illumination configuration. A 425-nm long-pass dichroic mirror (DMLP425, Thorlabs) was used to separate the excitation light and fluorescence emission. The fluorescence emission passed through the dichroic mirror and a 460±30  nm (MF460-60, Thorlabs) or 530±21.5  nm (MF530-43, Thorlabs) emission filter for imaging of NADH or FAD, respectively.^22^ A widefield image was formed on the 512×512 SPAD array camera (Lambert Instruments) with a tube lens (AC508-180-A, Thorlabs). A three-axis manual stage was used to translate the sample. Multispectral WF-FLIM of NADH and FAD channels was performed sequentially by manually sliding the filter mount insert. A 20-MHz modulation frequency (f), 301- to 1000-ms exposure time, and 25 phases were used per acquisition. Approximately 5 mW of power was used to illuminate the sample plane.

### 2.2 2D Cell Culture of Breast Cancer Cells

Following previously established cell culture methods,^30^ MDA-MB-231 (ER-/PR-/HER2-) and BT-474 (ER+/PR+/HER2+) cells were plated and expanded in monolayer culture to achieve the required cell numbers needed for xenograft tumor injection.

BT-474 cells: Upon thawing, BT-474 cells were cultured in a T-25 flask until they reached 75% to 80% confluency, after which they were passaged. The culture medium used for these cells was high-glucose Dulbecco’s modified Eagle medium, DMEM (ThermoFisher 11965092, Gibco) with 10% fetal bovine serum (FBS) and 1% penicillin: streptomycin. The confluent flasks were split into T-75 flasks by seeding 500,000 to 800,000 cells per flask. All cells were incubated at 37°C with 5% ${\mathrm{CO}}_{2}$ and 95% humidified air. Cell media were exchanged once every week after rinsing with PBS.

MDA-MB231 cells: Upon thawing, MDA-MB231 cells were cultured in a T-25 flask for a week until they reached 75% to 80% confluency. The culture medium used was RPMI 1640 (Gibco, 11879020) supplemented with 10% FBS, and 1% antibiotic-antimycotic, with an additional 0.5% glucose (Gibco™ A2494001). The confluent flasks were split into T-75 flasks by seeding 100,000 to 200,000 cells per flask. All cells were incubated at 37°C with 5% ${\mathrm{CO}}_{2}$ and 95% humidified air. Cell media were exchanged once every week after rinsing with PBS.

### 2.4 Tumor Generation

The animal studies were approved by the Texas A&M University Institutional Animal Care and Use Committee (IACUC 2024-0074). Tumors were generated in female athymic nude mice (J:NU, Jackson Laboratory) following previously published methods.^30^ Solutions of $10^{6}$ MDA-MB-231 or BT-474 cells in 100  μL of Matrigel were injected into the mammary fat pad. Tumors were allowed to grow to $∼200\;{\mathrm{mm}}^{3}$. Ten total mice were used, separated randomly into two groups of five mice for the two cell lines. A total of 30 tumors were generated (15 per cell line), as each mouse received three separate injections of cancer cells into three different mammary fat pads. Five control tissue samples were taken from untreated contralateral mammary fat pads.

### 2.5 System Validation

The optical imaging performance of the WF-FLIM microscope was first validated by imaging a grid distortion target (R1L3S3P, Thorlabs) and a negative 1951 USAF resolution target (R3L3S1N, Thorlabs) to characterize the field of view (FOV) and lateral resolution. The lateral resolution was quantified by taking a line intensity profile across a feature of the resolution target. A line intensity profile across a light-dark boundary measures the edge-spread function (ESF) of the microscope, and the first-order derivative of the ESF represents the line-spread function (LSF) of the system. The full-width at half maximum (FWHM) of the LSF describes the 2D point-spread function (PSF) and lateral spatial resolution of the WF-FLIM system.^31^

The fluorescence lifetime imaging capabilities of the system were validated with solutions of free and protein-bound NADH and free FAD. Solutions of free FAD in phosphate-buffered saline (PBS) were prepared at 10  μM, and solutions of free NADH in PBS were prepared at two concentrations: 50 and 5  μM. A protein-bound NADH solution (alpha = 0.6) was prepared by mixing 0.801 mL of lactate dehydrogenase (LDH) and 50  μM NADH in phosphate-buffered saline (PBS) to a total volume of 1 mL. Drops of 500  μL of the enzyme solutions were placed in a 35-mm glass-bottom imaging dish and lowered to the microscope focal plane by focusing on the edge of the drop.

To demonstrate the ability to image cellular autofluorescence for FLIM analysis, MDA-MB-231 and BT-474 breast cancer cells were plated at a density of $10^{6}\;\text{cells}$ per 35-mm glass-bottom imaging dishes (P35G-1.5-14-C, MatTek) 48 h before imaging. Cell media were replaced with PBS to remove background autofluorescence from the cell culture media. A total of nine locations were imaged for each cell line from three different dishes.

The clinical utility of this system was demonstrated by imaging freshly excised tissues from murine breast cancer tumor models, before sending the tissue for traditional histopathology. On the day of WF-FLIM tumor imaging, mice were humanely euthanized using isoflurane inhalation and cervical dislocation. Tumor and noninjected control mammary fat pad tissues were excised with a scalpel and forceps. Excised tissues were cut into $5\;{\mathrm{mm}}^{2}\times 5\;{\mathrm{mm}}^{2}$ sections, placed in a 35-mm imaging dish, and hydrated with drops of deionized (DI) water before WF-FLIM imaging. WF-FLIM imaging of each biopsy sample began within 30 min of tissue excision. At least three FOVs were acquired per tissue. After WF-FLIM imaging, tissues were fixed in 10% formalin and sent for standard histopathology analysis.

### 2.6 Image analysis

**Fig. 2:**
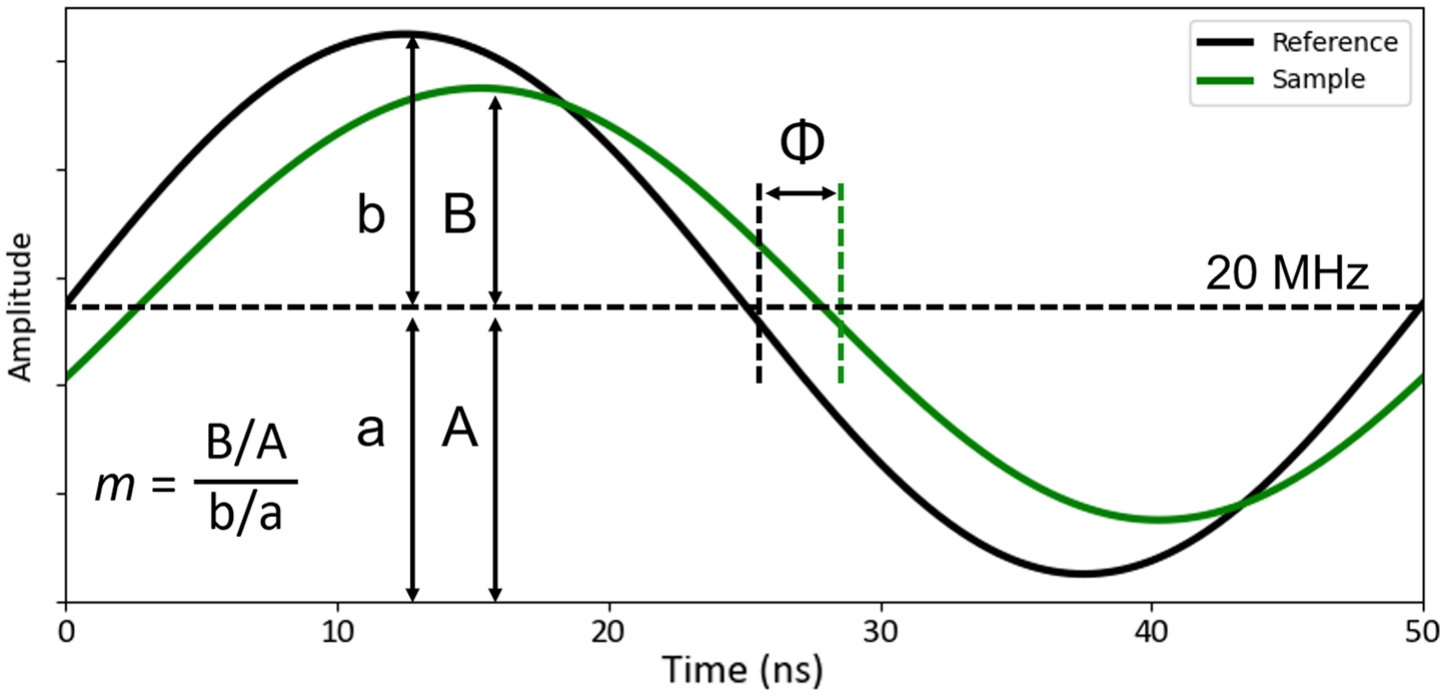
Frequency-domain fluorescence lifetime theory. A fluorophore with known fluorescence lifetime is used as the reference to calibrate the system and accurately quantify phase (Φ) and modulation (m) terms. Phase and modulation lifetimes are calculated using Eqs. (1) and (2).

The camera and illumination LED were controlled via a laptop with LIFA software (LIFA v2.0.0, Lambert Instruments). Following Lambert Instrument’s calibration protocol, the system was referenced by imaging a 4-μM solution of fluorescein (4.0 ns lifetime) to correct for the instrument frequency response and obtain accurate phase (Φ) and modulation (m) terms. The LIFA software used the sample and reference datasets to calculate and create normalized intensity, phase lifetime ($\tau_{\Phi }$), and modulation lifetime ($\tau_{m}$) images using Eqs. (1) and (2) and the angular modulation frequency (ω=2πf) (Fig. 2).^20^

$$\tan\;\Phi =\omega \tau_{\Phi },$$ (1)

$$m=\frac{1}{\sqrt{1+(\omega \tau_{m}{)}^{2}}.}$$ (2)

Normalized intensity images were created by flat-field correction; the sample intensity was divided by the reference intensity to correct for nonuniform illumination across the FOV. Whole-cell masks were created using Otsu’s thresholding applied to the normalized intensity images in MATLAB.^32^ Cell masks were then applied to the phase and modulation lifetime images. Reported lifetime values are presented as the mean ± standard deviation of the masked image.

### 2.7 Fluorescence Lifetime Sensitivity

To evaluate the FLIM sensitivity, a figure of merit, F-score, was calculated as

$$F=\frac{\Delta \tau }{\tau }/\frac{\Delta I}{I}.$$ (3)

Here, τ is the mean lifetime, Δτ is the lifetime standard deviation, I is the mean fluorescence intensity, and ΔI is the fluorescence intensity standard deviation.^33^^–^^35^ A lower F-score indicates a more sensitive and accurate lifetime calculation; a perfect FLIM system would achieve an F-score equal to 1.

### 2.8 Statistical Analysis

All statistical analysis was performed in RStudio. One-way ANOVA was conducted to compare the mean fluorescence lifetime between groups. Tukey-Kramer *post hoc* tests were performed to identify pairwise significant differences. An alpha level of 0.05 was used for all statistical tests. Data are presented as mean ± standard deviation unless otherwise noted. For tumor imaging, at least two unique FOVs were acquired and analyzed for each tissue sample.

## 3 Results

### 3.1 Spatial Resolution and Field of View

**Fig. 3:**
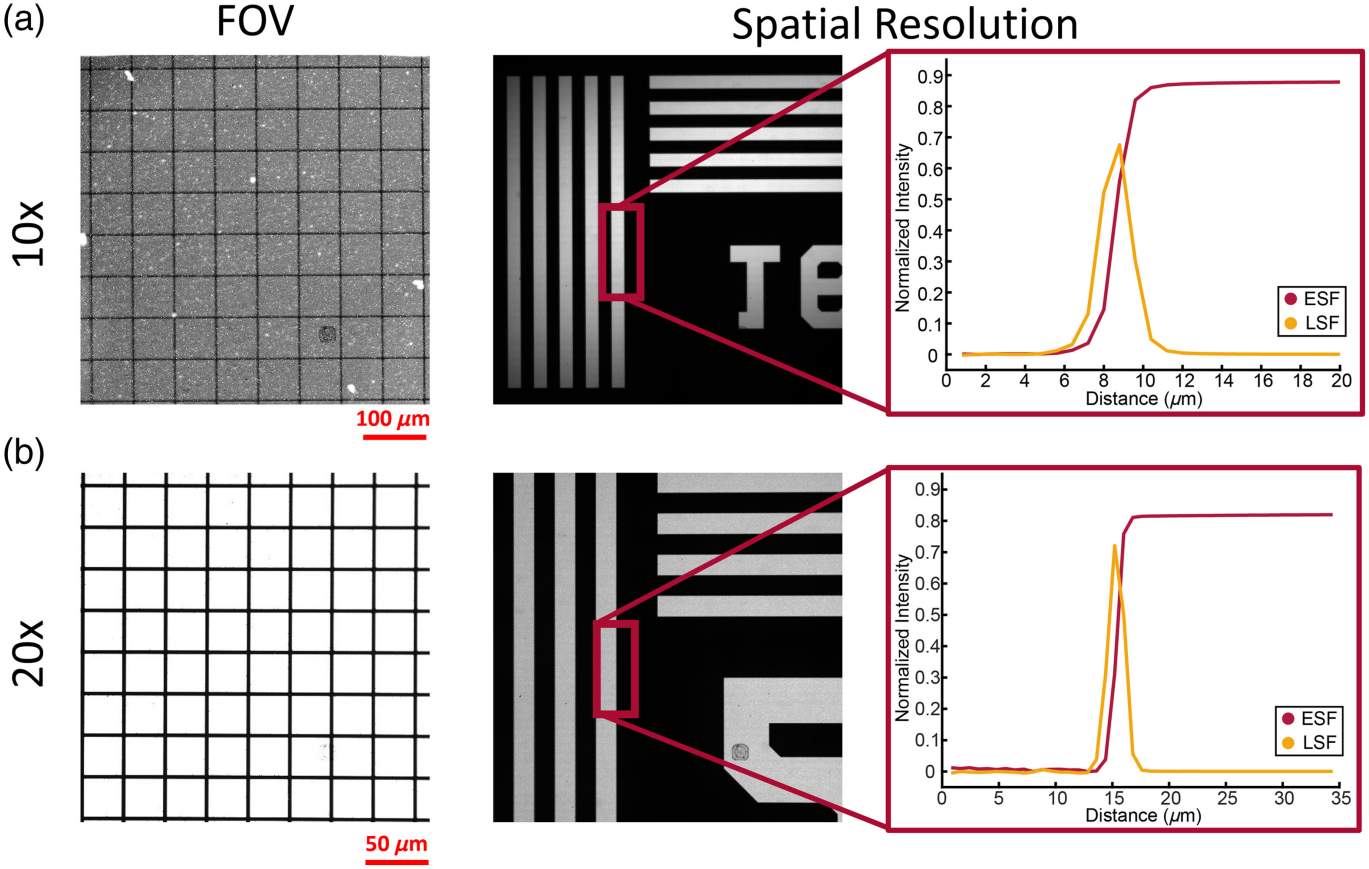
Optical performance characterization of WF-FLIM microscope with (a) 10× and (b) 20× objective lenses. The (a) 10× objective lens achieved a FOV and lateral spatial resolution of $800\;\mu m^{2}\times 800\;\mu m^{2}$ and 3.2  μm, respectively. The (b) 20× objective lens resulted in a $400\;\mu m^{2}\times 400\;\mu m^{2}$ FOV and 1.6  μm lateral spatial resolution.

The FOV and spatial resolution of the microscope were characterized by imaging a grid distortion target and 1951 USAF resolution target (Fig. 3). Using a 10× objective lens (i.e., overall 10× magnification), the FOV was $800\;\mu m^{2}\times 800\;\mu m^{2}$ with a lateral resolution of 1.92  μm [Fig. 3(a)]. Using a 20× objective lens, a $400\;\mu m^{2}\times 400\;\mu m^{2}$ FOV and 1.12  μm lateral resolution were achieved [Fig. 3(b)].

### 3.2 Multispectral Fluorescence Lifetime Validation

**Fig. 4:**
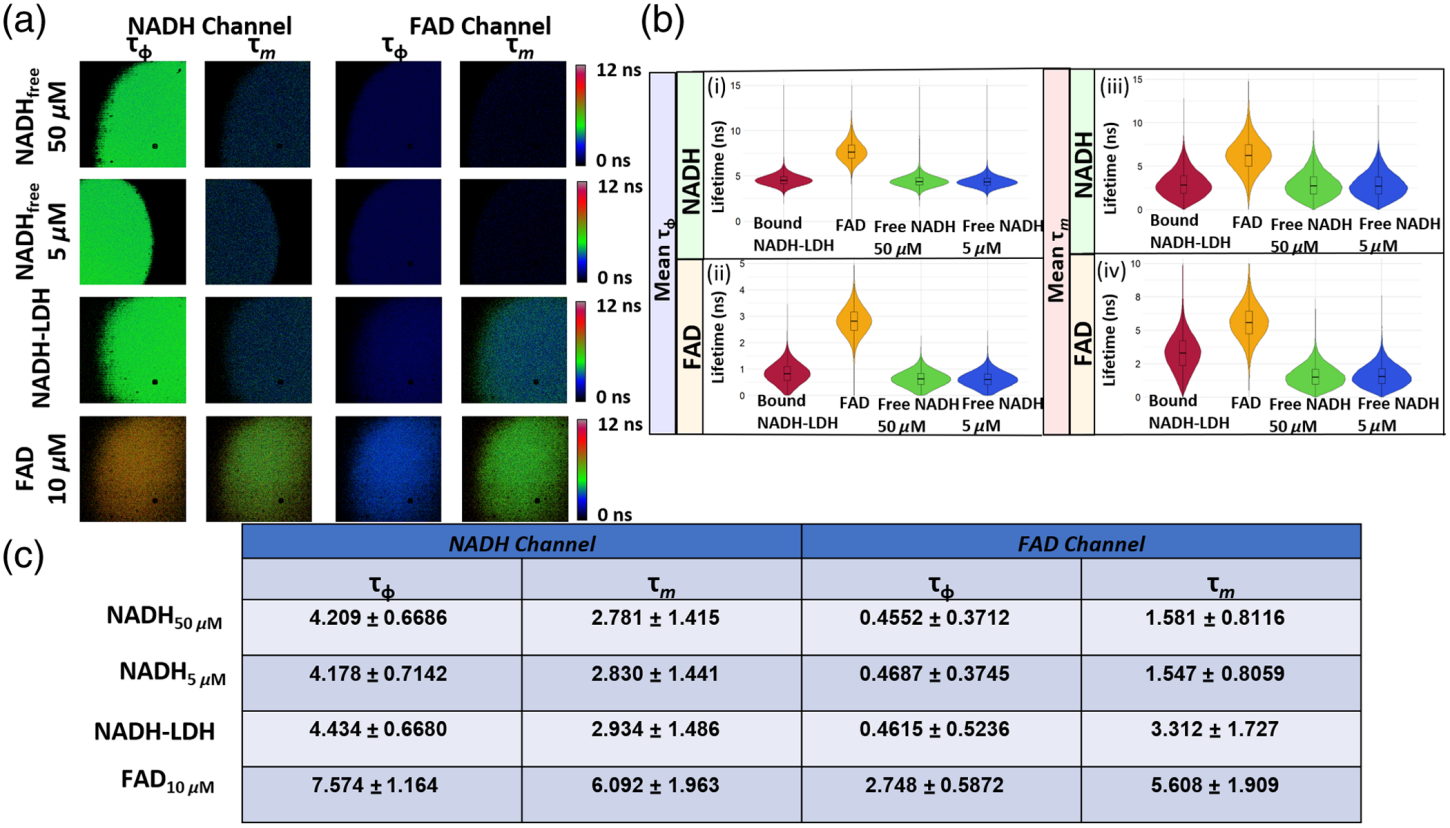
Characterization of fluorescence lifetime measurements of the WF-FLIM microscope. (a) Representative WF-FLIM images of solutions of free NADH, bound NADH-LDH, and free FAD. Image $\mathrm{FOV}=400\;\mu m^{2}\times 400\;\mu m^{2}$. (b) WF-FLIM resolves lifetime differences in solutions of free NADH, bound NADH-LDH, and free FAD. (c) Mean ± standard deviation fluorescence lifetime (ns) of WF-FLIM measured lifetimes of free NADH, bound NADH-LDH, and free FAD solutions.

To validate that the WF-FLIM system could faithfully measure the fluorescence lifetimes of endogenous metabolic coenzymes, solutions of NADH, in both free and protein-bound conformation states, and FAD were imaged (Fig. 4). Free NADH at 50 and 5  μM had near equivalent lifetimes; the percent difference between the two concentrations for the phase and modulation lifetimes were 0.74% and 1.75%, respectively. Protein-bound NADH showed an increase in both phase (5.95%) and modulation (3.61%) lifetimes, compared with 5  μM free NADH. FAD at 10  μM concentration had a 2.75±0.59  ns phase and 5.61±1.91  ns modulation lifetime measured from the FAD spectral channel. For imaging solutions of free NADH (50  μM), the phase and modulation F-scores were 2.335 and 7.480, respectively. For imaging solutions of FAD (10  μM), the phase and modulation lifetime F-scores were 1.427 and 3.059, respectively.

### 3.3 Metastatic (MDA-MB-231) and Nonmetastatic (BT-474) Cells in Monolayer

**Fig. 5:**
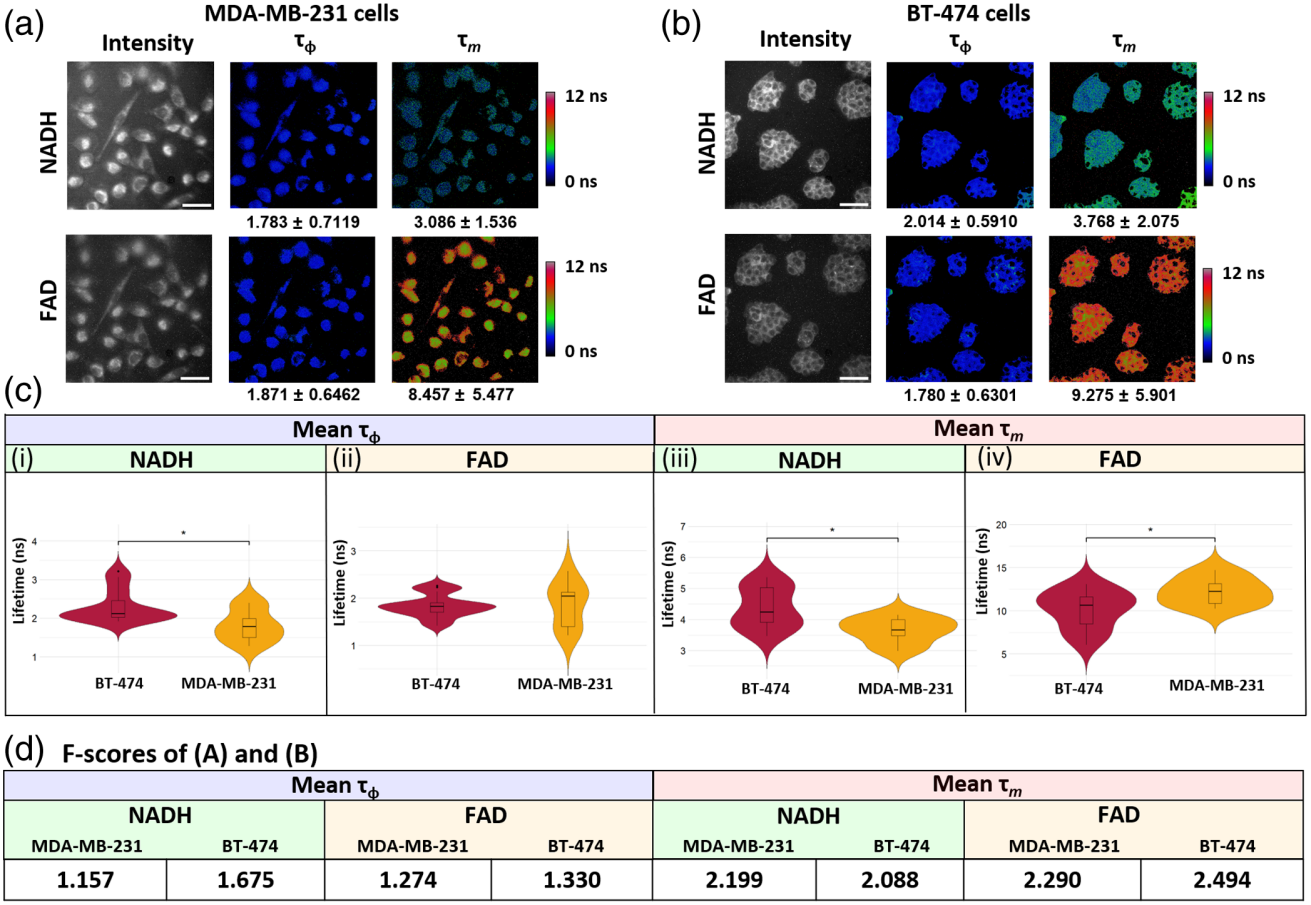
WF-FLIM microscopy of (a) MDA-MB-231. (b) BT-474 cells were plated on a 35-mm imaging dish. Cells were masked to quantify the mean lifetime ± standard deviation (ns) at the image level. Scale bar=100  μm. (c) WF-FLIM microscope can resolve temporal differences in fluorescence lifetime of MDA-MB-231 and BT-474 cells in a monolayer for both NADH (i–ii) and FAD (iii–iv) mean lifetimes. (d) F-scores for NADH and FAD phase and modulation lifetime imaging of MDA-MB-231 in (a) and BT-474 cells in (b).

To ensure the WF-FLIM microscope had sufficient spatial resolution and sensitivity to image cellular autofluorescence, breast cancer cells were plated in 35-mm dishes and imaged with the 20× objective lens configuration [Figs. 5(a)–5(b)]. The WF-FLIM microscope with 20× magnification achieved subcellular resolution, evident by the ability to visualize cell nuclei as dim structures in the raw intensity images [Figs. 5(a)–5(b)]. The mean ± standard deviation lifetime values, calculated at the image-level, are summarized below each masked lifetime image. The MDA-MB-231 cells displayed shorter NADH phase and modulation lifetimes than the BT-474 cells. The BT-474 cells showed slightly reduced FAD phase and modulation lifetimes than the MDA-MB-231 cells. Statistically significant differences between nonmetastatic BT-474 and metastatic MDA-MB-231 cell lines were found for the NADH phase, NADH modulation, and FAD modulation lifetime values (p-value<0.05) [Fig. 5(c)].

For MDA-MB-231 imaging, the NADH phase and modulation lifetime F-scores were 1.675 and 2.088, respectively. For MDA-MB231 imaging of FAD, the phase and modulation lifetime F-scores were 1.330 and 2.494, respectively. For BT-474 imaging, the NADH phase and modulation lifetime F-scores were 1.157 and 2.199, respectively. For BT-474 imaging of FAD, the phase and modulation lifetime F-scores were 1.274 and 2.290, respectively [Fig. 5(d)].

### 3.4 WF-FLIM of NADH and FAD in Biopsy Tumor Tissue Can Distinguish Between Nontumor and Tumor Tissues

**Fig. 6:**
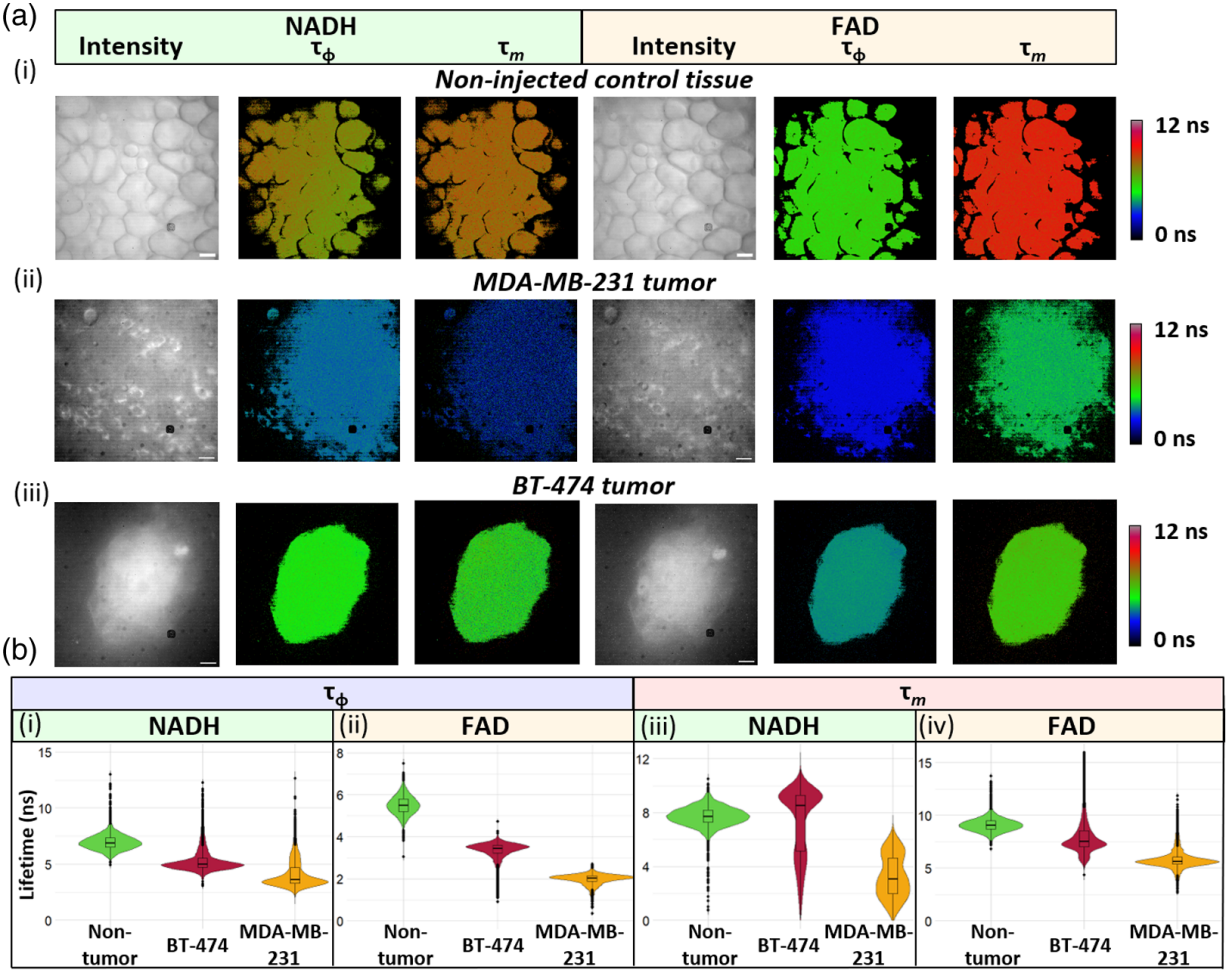
(a) Representative WF-FLIM images of (i) noninjected mammary fat pad, (ii) MDA-MB-231 tumor, and (iii) BT-474 tumor. Scale bar=50  μm. (b) WF-FLIM can resolve differences in fluorescence lifetime of noninjected mammary fat pad, MDA-MB-231 tumor, and BT-474 tumor using NADH and FAD lifetimes, with both phase (i–ii) and modulation lifetime values (iii–iv). Violin plots of the masked pixels in panel (a) represent the distribution of lifetime values within a tissue image.

To explore the system’s ability to image and ultimately identify cancer, we imaged and analyzed freshly excised tumor tissues and control (noninjected) mammary fat pad tissues. Representative control, MDA-MB-231 tumor, and BT-474 tumor tissues are shown [Fig. 6(a)]. Noninjected control tissue showed normal gross morphology of murine breast tissue with adipocytes clearly visible in both NADH and FAD intensity channels [Fig. 6(ai)]. In the fluorescence intensity images, MDA-MB-231 tumors displayed patchy growth and pronounced intensity variations; individual MDA-MB-231 cells could be resolved on the tissue surface using NADH and FAD intensity channels [Fig. 6(aii)]. BT-474 tumors, conversely, had slower and more confined growth and appeared as singular, cohesive masses [Fig. 6(aiii)]. Distributions of the NADH and FAD phase and modulation lifetimes in these representative FOVs show shorter lifetimes for MDA-MB-231 tumors than BT-474 tumors or control tissue [Fig. 6(b)].

**Fig. 7:**
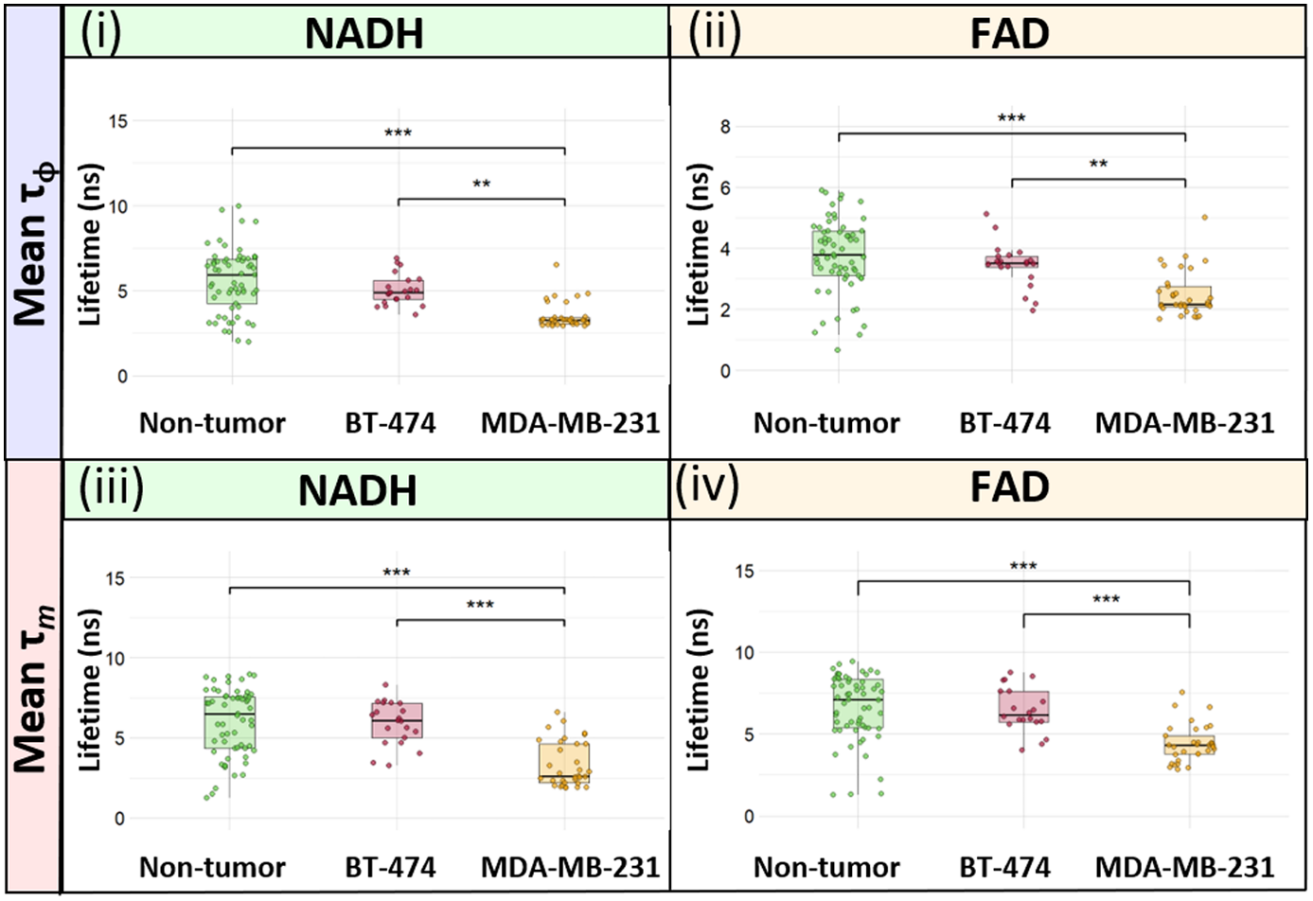
Pooled fluorescence lifetime analysis across all samples. Mean NADH and FAD phase (i–ii) and modulation (iii–iv) lifetimes are shown for noninjected mammary fat pad (nontumor), MDA-MB-231 tumor, and BT-474 tumor tissues. Each data point represents the mean fluorescence lifetime computed across each imaged location, n=32 images from nine nontumor tissues, n=27 images from nine BT-474 tumors, and n=47 images from ten MDA-MB-231 tumors.

**Fig. 8:**
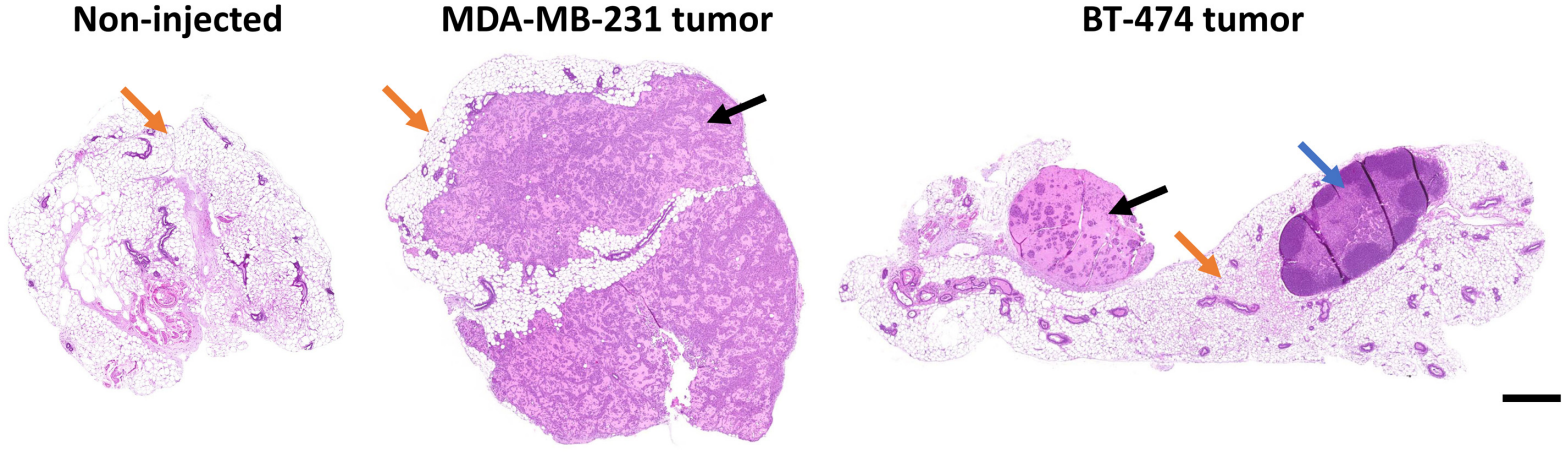
Representative histopathology H&E-stained images of noninjected control, MDA-MB-231 tumor, and BT-474 tumor tissues. Benign/normal breast tissue (orange arrows) is visually distinguishable from xenograft tumor tissue (black arrows) and lymph nodes (blue arrow) within H&E images. Scale bar=500  μm.

After imaging and histopathology diagnosis, each image was coded by the histopathology diagnosis of the tissue, resulting in three tissue groups: nontumor, BT-474 tumor, or MDA-MB-231 tumor tissue. Aggregated data of all images within the tissue types provide a comprehensive assessment of the fluorescence lifetime differences between nontumor, BT-474 tumor, and MDA-MB-231 tumor tissues (Fig. 7). In general, nontumor tissue had longer fluorescence lifetimes than both tumor tissue types (Fig. 7). Similarly, BT-474 tumor tissues had longer fluorescence lifetimes than MDA-MB-231 tumor tissues (Fig. 7). Histopathology confirmed tissue pathology (Fig. 8).

## 4 Discussion

Here, we demonstrated a desktop-sized FLIM system that could be used for POC cancerous tissue biopsy analysis. The WF-FLIM system was designed to interrogate autofluorescence from NADH (460±30  nm) and FAD (530±21.5  nm) using a single excitation wavelength (368 nm). These excitation and emission wavelengths were chosen based on the peak absorption and emission wavelengths of NADH and FAD.^22^ Murine xenograft tumors were imaged on the custom multispectral WF-FLIM system within 30 min of being excised. Sequential FOVs were imaged in a raster scan pattern; we did not notice photobleaching or phototoxicity effects during FLIM imaging. Further, we did not see any photodamage effects in the pathology images. Shorter NADH phase and modulation lifetimes were detected for cancerous compared with noncancerous tissues [Fig. 6(b)]. This agrees with previous studies that found a shorter NADH lifetime for MDA-MB231 breast cancer cells compared with MCF10a (nontumorigenic breast tissue cells) in both fresh and fixed samples.^36^ Similarly, the NADH phase and modulation lifetimes were shorter for metastatic MDA-MB-231 cells as compared with nonmetastatic BT-474 breast cancer cells (Figs. 5 and 6). This is also in agreement with literature-reported two-photon TSCPC-FLIM measured lifetime decreases in the mean NADH and FAD lifetimes of MDA-MB231 cells compared with nonmetastatic BT-474 cells in murine xenograft studies.^30^ Our results did not show shorter FAD lifetimes for monolayer cultures of MDA-MB231 compared with BT-474 cells, which have previously been reported with TCSPC-FLIM. This could be due to the low sensitivity of the FAD modulation lifetime channel, evident by large standard deviations and F-scores compared with the three other lifetime measurements.

Phase lifetime measurements demonstrated smaller standard deviations and more consistent trends among the NADH and FAD measurements, owing to the relatively higher insensitivity to the intensity fluctuations, compared with modulation lifetime measurements.^20^^,^^37^ Similarly, the NADH lifetime measurements showed smaller standard deviations than the FAD lifetime measurements. This discrepancy is likely due to a single excitation wavelength of 368 nm being used, which preferentially excites NADH. A more optimal excitation wavelength (i.e., 450 nm) for FAD could provide greater SNR in the FAD spectral channel and more accurate lifetime estimation.^22^ It is also possible that other endogenous fluorophores, such as collagen or lipofuscin, were contributing to the detected fluorescence signals. To improve the specificity of the system, future works could incorporate spectral unmixing and/or multiwavelength excitation.^38^

Other studies have also aimed to address the need for optical imaging-based pathological assessment at the POC. Microscopy with ultraviolet surface excitation (MUSE) can generate high-resolution images comparable with standard H&E histology imaging but require the use of exogenous fluorescent labels for contrast.^39^^–^^41^ NIR confocal laser endomicroscopy has been developed to identify cancer during biopsy procedures to better confirm diagnostic yield, but it also requires labeling with fluorescent drug-imaging agents.^42^ Raman spectroscopy and imaging have been applied for histopathological diagnosis of cancers, but these systems suffer from low signal intensity and long acquisition times and can require toxic metallic nanoparticles for signal enhancement.^43^^–^^45^ Other groups have similarly investigated FLIM for *ex vivo* tissue histopathological assessment; however, fiber-based systems have a fundamental compromise between FOV and imaging speed, limiting spatial resolution (2.73  μm) and FOV ($350\;{\mu m}^{2}\times 350\;{\mu m}^{2}$).^46^ Widefield FD-FLIM has recently been applied for NADH and protoporphyrin IX (PPIX) imaging in excised brain tumor specimens; however, PPIX fluorescence required administration of 5-aminolevulinic acid.^47^ Widefield TD-FLIM has been recently realized with the use of the emerging SPAD array detectors, but imaging speed and performance are still limited by weak autofluorescence signals and the number of pulses and temporal gates required for optimal sampling of the fluorescence decay curve.^27^

One advantage of our widefield imaging system is the relatively fast imaging speed. Here, a frame rate of 0.133 Hz (∼7.5  s/aFLIM image) was used, but prior work demonstrated imaging rates as fast as 1 Hz for aFLIM of breast cancer cell monolayers,^48^ allowing for full-field FLIM over areas of ∼800  μm×800  μm with subcellular spatial resolution. However, aFLIM imaging speed has historically been limited by the inherent low quantum yields of these endogenous fluorophores and detector pile-up and point-scanning image formation in TCSPC-based instruments.^49^ Here, the WF-FLIM signal-noise ratio (SNR) and imaging speed were similarly limited by weak autofluorescence signals. Specifically, the low intracellular concentration, low quantum yield, and short fluorescence lifetimes of NADH and FAD limit the number of detectable photons per pixel.

By contrast, MP-TCSPC systems can take minutes to acquire an image over a comparable FOV.^50^^,^^51^ Although MP-TCSPC can achieve F-scores as low as 1.24,^52^ this requires long pixel dwell times and overall image acquisition durations exceeding 30 s to a minute. Point-scanning image formation in MP-TCSPC is inherently slower than widefield, camera-based image acquisition, but enables higher illumination irradiance leading to increased photon emission rate and high SNR. Conversely, widefield and epi-illumination configurations result in relatively lower illumination irradiance and increased background fluorescence, requiring the use of relatively long exposure times to acquire enough photons for accurate lifetime quantification. Nonetheless, the parallel acquisition of 2D space by the SPAD array allows WF-FLIM to achieve comparatively fast FLIM imaging speeds. Incorporating optical sectioning via light-sheet illumination would produce a lower background signal and higher axial resolution and enable depth-resolved interrogation of suspicious tissue samples.^30^

MDA-MB-231 cells are an aggressive triple-negative breast cancer (TNBC) phenotype, whereas BT-474 cells are less aggressive HER2+ cells. The BT-474 cells displayed slower proliferation than MDA-MB231 cells and a tendency to clump and grow in aggregates. This was apparent in the WF-FLIM images of the monolayer culture (Fig. 5) and biopsied tumor (Fig. 6), and in the H&E histopathology image (Fig. 8). The differences in mean lifetimes between the two cancer cell lines highlight the ability of WF-FLIM of tissue autofluorescence to potentially identify distinct metabolic phenotypes of cells (Fig. 5) and tissues (Figs. 6 and 7). This study shows the utility of WF-FLIM at the POC for detecting and diagnosing cancer in biopsies of suspicious tissues. WF-FLIM could be extended to analyze fresh biopsies of the brain, lung, colon, liver, and other cancerous tissues.^46^^,^^53^^–^^55^ Owing to the imaging speed and relatively large FOV, this system could also be applied to intraoperative imaging of tissue margins.^56^

## 5 Conclusion

In summary, we report a desktop-sized widefield FD-FLIM system that can interrogate tissue autofluorescence lifetimes in a rapid, accurate, and label-free manner that does not interfere with traditional H&E pathology. This proof-of-concept study demonstrated the clinical utility of the system by imaging murine xenograft tumors within 30 min of biopsy and detecting differences in NADH and FAD phase and modulation lifetimes between noncancerous and cancerous tissues. Further, our WF-FLIM system was able to detect lifetime differences between two different subtypes of breast cancer, triple-negative breast cancer and HER2+ tumors.

## Data Availability

The data that support the findings of this article are not publicly available. Data are available from the authors upon request, and with permission from Caelum Diagnostic Solutions.
